# Acceptance of COVID-19 Vaccines among Patients with Inflammatory Bowel Disease in Japan

**DOI:** 10.3390/healthcare10010006

**Published:** 2021-12-22

**Authors:** Yu Nishida, Shuhei Hosomi, Yumie Kobayashi, Rieko Nakata, Masaki Ominami, Yuji Nadatani, Shusei Fukunaga, Koji Otani, Fumio Tanaka, Yasuaki Nagami, Koichi Taira, Noriko Kamata, Yasuhiro Fujiwara

**Affiliations:** Department of Gastroenterology, Osaka City University Graduate School of Medicine, Osaka 545-8585, Japan; m2076030@med.osaka-cu.ac.jp (Y.N.); yumie-koba@ktd.biglobe.ne.jp (Y.K.); m1310984@med.osaka-cu.ac.jp (R.N.); ominami@med.osaka-cu.ac.jp (M.O.); dada@med.osaka-cu.ac.jp (Y.N.); m1156849@med.osaka-cu.ac.jp (S.F.); kojiotani@med.osaka-cu.ac.jp (K.O.); m2079981@med.osaka-cu.ac.jp (F.T.); yasuaki-75@med.osaka-cu.ac.jp (Y.N.); koichit@med.osaka-cu.ac.jp (K.T.); m1266151@med.osaka-cu.ac.jp (N.K.); yasu@med.osaka-cu.ac.jp (Y.F.)

**Keywords:** surveys and questionnaires, severe acute respiratory syndrome coronavirus 2, mRNA-1273, BNT162b2, AZD1222

## Abstract

Coronavirus disease 2019 (COVID-19) vaccination is recommended for patients with inflammatory bowel disease (IBD). However, the acceptance of COVID-19 vaccines has not been sufficiently evaluated in patients with IBD. We aimed to assess the acceptance and hesitancy of COVID-19 vaccination and related factors among these patients. A retrospective cohort study using a self-reported questionnaire was performed among patients with IBD between 22 June 2021 and 30 August 2021. Of the 187 participants, 10.2% (*n* = 19) were hesitant to be vaccinated. Patients in the vaccine-hesitant group were younger (*p* = 0.009) and had a shorter disease duration (*p* = 0.020). Vedolizumab was prescribed more frequently (*p* = 0.024) and immunomodulators were less frequently used (*p* = 0.027) in this group. Multivariable logistic regression analysis identified age (odds ratio [OR]: 0.96, 95% confidence interval [CI]: 0.92–1.00, *p* = 0.042) and the use of immunomodulators (OR: 0.08, 95% CI: 0.01–0.66, *p* = 0.019) as independent significant factors for vaccine hesitancy. The COVID-19 vaccine hesitancy rate in patients with IBD in Japan was 10% in this study. The Japanese COVID-19 vaccination campaign appears to be successful. The risk of COVID-19 among patients with IBD requires adequate measures to ensure that vaccines are accepted by vaccine-hesitant patients. These findings may be helpful in achieving adequate vaccination rates.

## 1. Introduction

Coronavirus disease 2019 (COVID-19), caused by severe acute respiratory syndrome coronavirus 2 (SARS-CoV-2), emerged in Wuhan, China, in December 2019 and the outbreak rapidly spread worldwide [1]. Vaccines are authorized and recommended to prevent COVID-19 because of their high safety and efficacy [2,3]. The presence of a vaccine is a key element to prevent COVID-19, and therefore, to minimize new infections. It is crucial to vaccinate people to prevent the spread of COVID-19 [3]. In Japan, the COVID-19 vaccination campaign for older individuals started in April 2021, followed by people with underlying diseases, and then the rest of the general population. At the time of this survey, three vaccines against COVID-19 have been approved in Japan: mRNA-1273 (Moderna, Cambridge, MA, USA), BNT162b2 (Pfizer/BioNTech, New York, NY, USA), and AZD1222 (AstraZeneca, Cambridge, UK). However, vaccine hesitancy remains high even in the COVID-19 pandemic period [4]. The COVID-19 vaccine hesitancy rate was reported to be 20% (13–29%) among ordinary people in a systematic review [5]. In Japan, 11.0 to 12.3% of the general population are unwilling to be vaccinated [6,7,8]. Previous studies have reported that COVID-19 vaccine hesitancy is associated with sex, young age, living alone, low income, presence of severe psychological distress, region, intention to be vaccinated against influenza in the past year, and a low education level [5,8,9,10,11,12,13,14].

Inflammatory bowel disease (IBD), namely ulcerative colitis and Crohn’s disease, are intestinal disorders [15,16,17,18,19]. During the course of the disease, the majority of the patients with IBD need long-term immunomodulatory therapies and thus, have an increased risk of infectious diseases [20,21]. Patients with IBD are influenced by the lifestyle changes caused by the appearance of COVID-19 [22,23].

A low acceptance rate of vaccination was reported in a systematic review among patients with IBD, ranging from 11% to 54% [24]. Regarding COVID-19, IBD itself does not increase the risk of developing the disease [25]. Therefore, clinical guidelines recommend that patients should continue current IBD medications [26,27]. The incidence of adverse events of special interest in IBD patients after COVID-19 vaccination was low and similar to that of a matched cohort of patients without IBD [28]. This includes patients on immunosuppressive agents. The benefits of COVID-19 vaccination in patients with IBD probably outweigh the minimal risk [28]. Furthermore, current studies suggested that systemic corticosteroids might increase the risk of hospitalization, as well as the risk of requiring ventilation, intensive care unit care, and death among patients with immune-mediated inflammatory disease [29,30]. Patients with IBD are at risk of using steroids, which may lead to aggravation of COVID-19. Therefore, vaccination of patients with IBD should be a higher priority than vaccination of members of the general public. The International Organization for the Study of Inflammatory Bowel Disease and British Society of Gastroenterology recommended vaccinating all patients with IBD as soon as they were able to receive the vaccination, regardless of immune-modifying therapies [31,32]. The International Organization for the Study of Inflammatory Bowel Disease and British Society of Gastroenterology stated that the benefits of vaccination are likely to outweigh these theoretical concerns, even in patients treated with antitumor necrosis factor (TNF) drugs, and that the risks of COVID-19 vaccination in patients with IBD are anticipated to be very low. They recommend that patients with IBD accept whichever approved COVID-19 vaccination is offered to them. Immunosuppressive therapies have been reported to reduce the effectiveness of vaccines. Immunomodulatory therapies, which are often used for IBD patients, have been reported to result in an attenuated vaccine response [33,34,35]. Vedolizumab is an anti-integrin monoclonal antibody directed against α4β7 integrin. It has a low incidence rate of serious infection, and it would not affect the vaccine response in healthy controls [36,37]. Anti-SARS-CoV-2 antibody concentrations were higher in patients with vedolizumab therapy compared with patients with infliximab therapy who received COVID-19 vaccination [38]. The combination of anti-TNF with immunomodulators (azathioprine, 6-meracptopurine, methotrexate) resulted in an attenuated vaccine response as compared to anti-TNF monotherapy [33].

Regarding COVID-19 vaccinations among patients with IBD, 17.7% were hesitant to be vaccinated in an Italian survey [39], 24.0% in a French survey [40], and 14% in a Spanish survey [41]. Some studies have reported the acceptance of COVID-19 vaccines; however, different studies have had conflicting results, and no studies have reported the acceptance of COVID-19 vaccines in Japan [42]. Consequently, there is an urgent need for an understanding of current attitudes toward vaccines and factors determining vaccine intent in patients with IBD in Japan.

Therefore, we aimed to explore hesitancy toward, and acceptance of, COVID-19 vaccines in patients with IBD and to identify factors associated with these attitudes, including consideration of participants’ background, lifestyle, disease condition, medication, and the presence of other people in the household.

## 2. Materials and Methods

### 2.1. Study Design and Participants

This was a retrospective cohort study using a questionnaire among patients with IBD (≥16 years old) who attended regular follow-up at Osaka City University Hospital from 22 June 2021 to 30 August 2021. We asked patients with IBD who visited our hospital to voluntarily answer the questionnaire. The data were collected anonymously.

### 2.2. Exclusion Criteria

The exclusion criteria were a diagnosis of IBD within the previous 3 months; an inability to complete the questionnaire, despite assistance; and lack of consent.

### 2.3. Questionnaire Design

A self-administered questionnaire was developed based on past literature on similar topics [42,43,44]. The questionnaire included questions regarding the patient’s demographic data (sex, age at recruitment, and age at disease diagnosis), epidemiological history of COVID-19, gastrointestinal symptoms, current medication use, history of COVID-19 vaccination, intention to be vaccinated against COVID-19, and reasons for acceptance or refusal of such vaccination (Table S1). Additionally, the questionnaire included questions about factors that have been reported to be associated with COVID-19 vaccination hesitancy, such as cohabitation status, smoking status, and comorbidities [5,8,9,10,11,12]. After providing online consent, participants completed several questionnaires using the secure Research Electronic Data Capture (REDCap) platform. Participants used a personal computer or smart phone with a complete survey [45] and completed the survey at their convenience so that they could answer the questionnaire without worrying about time.

### 2.4. Ethical Considerations

All participants had to provide their consent before they proceeded to the questionnaire response page, and the Ethics Review Board of Osaka City University Graduate School of Medicine approved this study (no. 2021-102). The questionnaires were made anonymous to ensure patient privacy.

### 2.5. Statistical Analysis

Continuous variables are summarized as medians and interquartile ranges. Clinical characteristics between the vaccinated and those not intending to be vaccinated were compared using either the chi-square test or Fisher’s exact test for categorical variables and the Mann–Whitney U-test for continuous variables. We used univariate logistic regression analyses to calculate unadjusted odds ratios of factors associated with vaccine hesitancy. Further, those factors presumed to be risk factors for vaccine hesitancy were then quantified using multivariable analysis with factors exhibiting statistical significance in the univariate analysis and factors reported to be associated with vaccine hesitancy.

Statistical significance was set at *p* < 0.05. All statistical analyses were performed with EZR (Saitama Medical Center, Jichi Medical University), a graphical user interface for R (The R Foundation for Statistical Computing, version 2.13.0).

## 3. Results

### 3.1. Study Participants

During this period, 187 patients with IBD were included. The demographic and IBD characteristics of the study population are presented in Table 1.

### 3.2. Assessment of COVID-19 Vaccination Hesitancy

**Figure 1:**
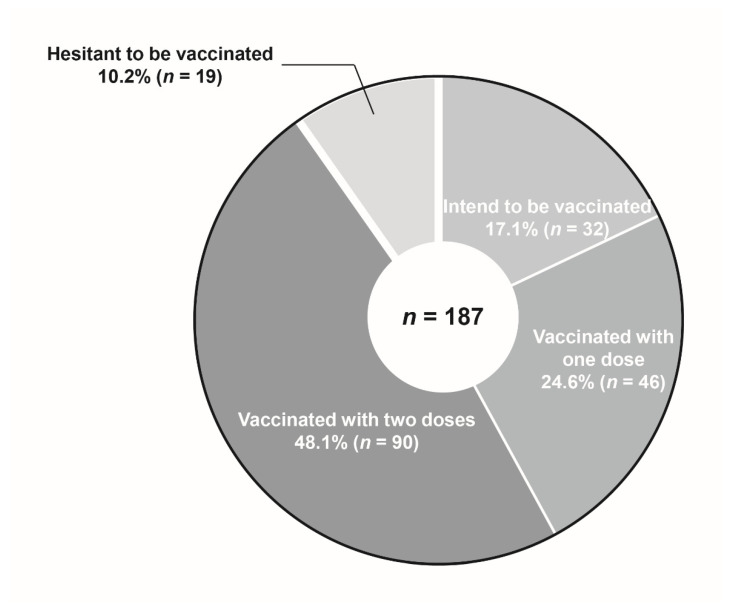
Willingness and hesitancy of patients with inflammatory bowel disease to receive a coronavirus disease 2019 vaccine.

Of the participants, 17.1% (*n* = 32) intended to be vaccinated, 24.6% (*n* = 46) had been vaccinated once, 48.1% (*n* = 90) had completed two vaccinations, and 10.2% (*n* = 19) were hesitant to be vaccinated (Figure 1).

### 3.3. Factors Associated with Vaccine Hesitancy

Patients in the vaccine-hesitant group were younger (*p* = 0.009) and had shorter IBD disease duration (*p* = 0.020). Patients who were hesitant to be vaccinated were more likely to be on treatment with vedolizumab (*p* = 0.024) and were less likely to be using immunomodulators (azathioprine or 6-mercaptopurine) (*p* = 0.027). Patients who were willing to be vaccinated were more likely to live with an older person (Table 1).

The patients’ sociodemographic factors were analyzed to identify factors associated with vaccine hesitancy. From the univariate logistic regression analysis, vaccine hesitancy exhibited a significant relationship with age (OR: 0.96, 95% CI: 0.92–0.99, *p* = 0.011), the use of immunomodulators (OR: 0.13, 95% CI: 0.02–0.98, *p* = 0.048), and the use of vedolizumab (OR: 7.69, 95% CI: 1.58–37.40, *p* = 0.012). Multivariable logistic regression analysis was performed to identify the factors related to vaccine hesitancy. Variables in the multivariate analysis were selected among factors exhibiting statistical significance in the univariate analysis and factors reported to be associated with vaccine hesitancy: sex, age at enrollment, disease duration, living with older individuals, use of immunomodulators and use of vedolizumab [5,8,9,10,11,12,13,14]. In the multivariable analysis, age (OR: 0.96, 95% CI: 0.92–1.00, *p* = 0.042) and the use of immunomodulators (OR: 0.08, 95% CI: 0.01–0.66, *p* = 0.019) was significantly associated with vaccine hesitancy (Table 2). Younger age was positively associated with COVID-19 vaccine hesitancy, whereas the use of immunomodulators was negatively associated with vaccine hesitancy.

### 3.4. Reasons for COVID-19 Vaccine Hesitancy among Patients with IBD

Among patients with IBD, COVID-19 vaccine hesitancy was due to concerns about the long-term safety of the vaccines (*n* = 7), possible adverse interaction with immunosuppressive therapies (*n* = 7), lack of trust regarding vaccine development or the testing process (*n* = 3), and concerns about short-term adverse reactions (*n* = 2) (Table 3). The reasons for accepting COVID-19 vaccines are summarized in Table 4.

## 4. Discussion

### 4.1. Hesitancy and Acceptance of OCIVD-19 in Patients with IBD

The acceptance of patients with IBD to be vaccinated against COVID-19 has not been sufficiently evaluated and the reason for COVID-19 vaccine hesitancy among patients with IBD has not been well understood. We analyzed data from 187 patients with IBD to identify their COVID-19 vaccination intentions and their rationale. We found that 10.2% of patients with IBD were hesitant to get vaccinated against COVID-19, and the significantly associated factors in the adjusted analysis were the use of immunomodulators and younger age.

Regarding the use of immunomodulators, patients who were not taking immunomodulators might be more likely to be hesitant to use the vaccine. Among the 19 patients in the vaccine-hesitant group, seven patients replied that this was due to their receiving immunosuppressive therapies (Table 3). Among these seven patients, none received immunomodulators, three received corticosteroids, two received TNF therapies, and two received vedolizumab. Patients hesitant to receive vaccines due to immunosuppressive states might think that immunosuppressive drugs could reduce the effectiveness of the vaccines. They might misunderstand that the COVID-19 vaccine was contraindicated, similar to live vaccines. However, COVID-19 vaccination is recommended even for patients with IBD receiving immunosuppressive therapies [31,32]. Of the 187 patients in the vaccine acceptance group, 13 patients replied that the acceptance of vaccination was due to receiving immunosuppressive therapies. However, the number of participants with immunomodulators is very limited and further research would be needed to conclude this. Physicians should inform patients about the necessity of vaccination with immunomodulators and should alleviate their fears.

In contrast, the use of vedolizumab would be a confounding factor for vaccine hesitancy. Vedolizumab was prescribed more frequently in the hesitant group (*p* = 0.024) and from the univariate logistic regression analysis, vaccine hesitancy exhibited a significant relationship with the use of vedolizumab (OR: 7.69, 95% CI: 1.58–37.40, *p* = 0.012). However, patients with vedolizumab therapy were significantly younger compared with patients without vedolizumab therapy (*p* = 0.037) and multivariable logistic regression analysis did not identify the use of vedolizumab as an independent factor for vaccine hesitancy.

The younger generation has been reported to be more likely to refuse the vaccine [7,9]. This is in accordance with the results of this study. Galle et al. [46] reported that age and sex were not significantly associated with vaccine acceptance in the multivariable analysis. The lack of association with age was probably because the age range of their study sample was too narrow to detect an age difference. Several studies have reported factors related to vaccine hesitancy, such as age, sex, income, and education [5,6,43]. In this study, we could not evaluate the influence of income and education on vaccine acceptance because the questionnaire did not include questions on these factors. Regarding sex, no statistically significant differences in vaccine hesitancy were noted between males and females, but this may be due to the small sample size.

It is unclear whether patients with IBD are more or less willing to be vaccinated than members of the general population. In one study, patients with IBD were reported to be significantly more hesitant to be vaccinated than control patients without IBD [47]; however, in another study, Dalal et al. [42] reported that willingness to be vaccinated against COVID-19 was higher in patients with IBD than expected based on surveys of the general population in the United States. Our findings suggest that the proportion of patients with IBD who are hesitant about vaccination against COVID-19 vaccine is similar to that of the general population, as reported in different Japanese studies [6,7,8]. This prevalence of vaccine hesitancy in this study was relatively low compared with that in previous reports of patients with IBD [40,42]. This could be due to the timing of the study because COVID-19 has become recognized as a long-term issue and the high effectiveness and safety of vaccination have been widely recognized, although intentions vary widely according to the characteristics of the patient sample, country, and time period [6,48,49,50].

The majority of patients with severe COVID-19 have at least one comorbidity [51]. Specific risk factors for severe COVID-19 include older age, diabetes, cardiovascular disease, chronic kidney disease, and obstructive pulmonary disease [52]. IBD has not been reported to be associated with an increased risk of severe COVID-19; however, Brenner et al. [53] reported that a higher proportion of patients with IBD who were on steroid treatment required mechanical ventilation or intensive care unit admission and that patients on steroids had higher mortality than patients on other medications. Patients with IBD, who are more likely to require treatment with steroids, should be especially careful to avoid SARS-CoV-2 infection. The long-term side effects of the mRNA vaccines are not yet known; however, achieving an adequate COVID-19 vaccination rate is essential to avoid SARS-CoV-2 infection in patients with IBD. Patients, especially young patients and those receiving immunosuppressive therapies, need to be informed of the importance of vaccination. Doctors’ recommendations and explanations may assist patients who are hesitant to be vaccinated against COVID-19 to be more receptive to COVID-19 vaccination.

### 4.2. Study Limitations

This study has some limitations including its single-center nature and relatively small sample size. First, this study may have had some selection bias because it was conducted in a single tertiary center. Study participants may have had more complicated disease than IBD patients in general. However, we perform medical follow-up of patients even if they are in remission, so this is unlikely to have had a major effect on the generalizability of the findings. There may also have been participation (self-selection) bias. The prevalence of vaccine hesitancy may have differed among patients who declined to participate. Furthermore, this questionnaire did not include other factors, such as daily habits, educational level, or income [6,7,8], as this was made to be simple and easy to answer. Additionally, as a cross-sectional design was used, a causal relationship could not be inferred.

### 4.3. Future Research

Despite these limitations, our findings are unique in that they are the first to report on COVID-19 vaccine intent among patients with IBD in Japan. Appropriate instructions are needed for patients with IBD especially in young patients and patients using immunosuppressive drugs. Physicians’ assistance may be required to improve the vaccination rate among patients with IBD. Vaccination rates are rising worldwide but have already peaked in many countries and additional measures may be needed to further increase vaccination coverage.

## 5. Conclusions

### 5.1. Acceptance of COVID-19 Vaccine

This is the first study to access COVID-19 vaccine hesitancy among patients with IBD in Japan. The acceptance of COVID-19 vaccine in patients with IBD in Japan was relatively high compared to that reported in previous studies. This study indicates factors associated with COVID-19 acceptance were older age and the use of immunomodulators (azathioprine or 6-mercaptopurine). However, this study design had a single-center nature and a relatively small sample size. Further study would be needed to conclude this.

### 5.2. Practical Implications

The theoretical safety of the. mRNA vaccine during immunomodulator use, evidence-based safety, and patient education with correct information could contribute toward the improvement of the vaccination rate. It is necessary to explain to patients that the benefits of vaccines outweigh the disadvantages, especially in patients without immunomodulators and young patients. Further efforts should be made to protect patients with IBD from SARS-CoV-2 infection and to achieve adequate vaccination coverage.

## Figures and Tables

**Table 1:** Sociodemographic characteristics of the study participants by COVID-19 vaccination intention.

	All Patients	Willing to Be Vaccinated	Hesitant to Be Vaccinated	*p*-Value
Demographics				
Number of patients	187	168	19	
Disease type				0.221
Ulcerative colitis	107 (57.2%)	99 (58.9%)	8 (42.1%)	
Crohn’s disease	80 (42.8%)	69 (41.1%)	11 (57.9%)	
Sex				0.454
Male	120 (64.2%)	106 (63.1%)	14 (73.7%)	
Female	67 (35.8%)	62 (36.9%)	5 (26.3%)	
Age at enrollment (years), median (IQR)	45.0 (32.5–56.5)	46.0 (34.8–58.3)	32.0 (29.5–43.5)	0.009
Age at diagnosis (years), median (IQR)	26.0 (20.0–40.0)	26.0 (20.0–40.0)	24.0 (19.0–27.5)	0.185
Disease duration (years), median (IQR)	13.0 (6.0–22.0)	13.0 (7.0–23.3)	6 (5–13.5)	0.020
Smoking				0.324
Yes	31 (16.5%)	26 (15.5%)	5 (26.3%)	
No	156 (83.4%)	142 (84.5%)	14 (73.7%)	
Drinking				0.801
Yes	82	73	9	
No	105	95	10	
Occupation				
Worker	97 (51.9%)	86 (51.2%)	11 (57.9%)	0.634
Office worker	64 (34.2%)	59 (35.1%)	5 (26.3%)	0.611
Health worker	13 (7.0%)	10 (6.0%)	3 (15.8%)	0.132
Sales assistant	4 (2.1%)	4 (2.4%)	0 (0%)	>0.999
Education	3 (1.6%)	3 (1.8%)	0 (0%)	>0.999
Delivery person	3 (1.6%)	3 (1.8%)	0 (0%)	>0.999
Housemaker	26 (13.9%)	24 (14.3%)	2 (10.5%)	>0.999
Student	10 (5.3%)	8 (4.8%)	2 (10.5%)	0.269
Cohabitation				
Living with older individuals	54 (28.9%)	52 (31.0%)	2 (10.5%)	0.067
Living with children	58 (31.0%)	53 31.5(%)	5 26.3(%)	0.796
Living with persons with comorbidities	45 (24.1%)	42 (25.0%)	3 (15.8%)	0.572
Comorbidity				
Hypertension	23 (12.3%)	23 (13.7%)	0 (0%)	0.136
Cardiac disorder	0 (0%)	0 (0%)	0 (0%)	···
Chronic Obstructive Pulmonary Disease	0 (0%)	0 (0%)	0 (0%)	···
Diabetes mellitus	4 (2.1%)	4 (2.4%)	0 (0%)	>0.999
Bronchial asthma	2 (1.1%)	2 (1.2%)	0 (0%)	>0.999
Hepatic disorder	5 (2.7%)	5 (3.0%)	0 (0%)	>0.999
Renal disorder	7 (3.7%)	7 (4.2%)	0 (0%)	>0.999
Psychiatric disorder	16 (8.6%)	15 (8.9%)	1 (5.3%)	>0.999
Medication				
Mesalamine	127 (67.9%)	113 (67.3%)	14 (73.7%)	0.796
Corticosteroids	18 (9.6%)	15 (8.9%)	3 (15.8%)	0.402
Immunomodulators (azathioprine or 6-mercaptopurine)	52 (27.8%)	51 (30.4%)	1 (5.3%)	0.027
Anti-TNF	50 (26.7%)	44 (26.2%)	6 (31.6%)	0.593
Ustekinumab	23 (12.3%)	19 (11.3%)	4 (21.1%)	0.261
Vedolizumab	7 (3.7%)	4 (2.4%)	3 (15.8%)	0.024
Tofacitinib	4 (2.1%)	4 (2.4%)	0 (0%)	>0.999
COVID-19				
COVID-19 contact	4 (2.1%)	4 (2.4%)	0 (0%)	>0.999
COVID-19 isolation	4 (2.1%)	4 (2.4%)	0 (0%)	>0.999
COVID-19 infection	2 (1.1%)	2 (1.2%)	0 (0%)	>0.999

COVID-19, coronavirus disease 2019; IQR, interquartile range; TNF, tumor necrosis factor.

**Table 2:** Univariate and multivariable logistic regression analysis of hesitancy toward COVID-19 vaccination.

	Unadjusted OR (95% CI)	*p*-Value	Adjusted OR (95% CI)	*p*-Value
Demographics				
Disease type				
Ulcerative colitis	Ref.			
Crohn’s disease	1.97 (0.75–5.16)	0.166		
Sex				
Male	Ref.		Ref.	
Female	0.61 (0.21–1.78)	0.365	0.38 (0.11–1.31)	0.126
Age at enrollment	0.96 (0.92–0.99)	0.011	0.96 (0.92–1.00)	0.042
Age at diagnosis	0.97 (0.93–1.01)	0.171		
Disease duration	0.95 (0.90–1.00)	0.056	0.98 (0.92–1.05)	0.477
Smoking				
No	Ref.			
Yes	1.95 (0.65–5.88)	0.235		
Alcohol consumption				
Non-drinker	Ref.			
Drinker	0.71 (0.15–3.25)	0.655		
Occupation				
Worker				
No	Ref.			
Yes	1.31 (0.50–3.42)	0.580		
Office worker				
No	Ref.			
Yes	0.66 (0.23–1.92)	0.446		
Health worker				
No	Ref.			
Yes	2.96 (0.74–11.90)	0.125		
Housemaker				
No	Ref.			
Yes	0.71 (0.15–3.25)	0.655		
Student				
No	Ref.			
Yes	2.35 (0.46–12.0)	0.303		
Cohabitation				
Living with older individuals				
No	Ref.		Ref.	
Yes	0.26 (0.06–1.19)	0.081	0.25 (0.05–1.25)	0.091
Living with children				
No	Ref.			
Yes	0.77 (0.27–2.27)	0.641		
Living with persons with comorbidities				
No	Ref.			
Yes	0.56 (0.16–2.04)	0.379		
Medication				
Mesalamine				
No	Ref.			
Yes	1.36 (0.47–3.98)	0.571		
Corticosteroids				
No	Ref.			
Yes	1.91 (0.50–7.32)	0.344		
Immunomodulators (azathioprine or 6-mercaptopurine)				
No	Ref.		Ref.	
Yes	0.13 (0.02–0.98)	0.048	0.08 (0.01–0.66)	0.019
Anti-TNF therapy				
No	Ref.			
Yes	1.30 (0.47–3.63)	0.616		
Ustekinumab				
No Yes	Ref.			
	2.09 (0.63–6.96)	0.229		
Vedolizumab				
No	Ref.		Ref.	
Yes	7.69 (1.58–37.40)	0.012	4.29 (0.67–27.50)	0.124
Tofacitinib				
No	Ref.			
Yes	5.51e-7 (0.00–Inf)	0.990		

CI, confidence interval; COVID-19, coronavirus disease 2019; Inf., infinity; IQR, interquartile range; Ref., reference; TNF, tumor necrosis factor.

**Table 3:** Reasons for COVID-19 vaccination hesitancy.

Reasons for Hesitating to Get Vaccinated	*n* (%)
Concerned that Long-Term Safety of Vaccines is Unknown	7 (36.8%)
Receiving immunosuppressive therapy	7 (36.8%)
Lack of trust regarding vaccine development or testing process	3 (15.8%)
Concerned about short-term adverse reaction	2 (10.5%)

COVID-19, coronavirus disease 2019.

**Table 4:** Reasons for the acceptance of COVID-19 vaccination.

Reasons for Wanting to Be Vaccinated	*n* (%)
Acquiring immunity against COVID-19	77 (45.8%)
Protecting others from COVID-19	42 (25.0%)
Increased risk of severe COVID-19 due to old age or comorbidities	21 (12.5%)
Receiving immunosuppressive therapy	13 (7.7%)
Desire to return to normal life	8 (4.8%)
Acquiring herd immunity	4 (2.4%)
Recommendation from physician	2 (1.2%)
Low incidence of adverse effects	1 (0.6%)

COVID-19, coronavirus disease 2019.

## Data Availability

The data presented in this study are available on request from the corresponding author. The data are not publicly available due to privacy restrictions.

## Supplementary Materials

The following are available online at https://www.mdpi.com/article/10.3390/healthcare10010006/s1, Table S1: questionnaire.
