# Supplementary material for: Acceptance of COVID-19 Vaccines among Patients with Inflammatory Bowel Disease in Japan
Source: Healthcare (Basel). 2021 Dec 22;10(1):6. doi: 10.3390/healthcare10010006 (PMC8774988; doi:10.3390/healthcare10010006)
Supplement: Supplementary file 1 [file healthcare-10-00006-s001.zip › healthcare-1472418-supplementary.pdf]

healthcare

MDPI

Table S1. questionnaire.

- Gender
- ☐ M
- ☐ F
- Age

___

- What is your current occupation?
- ☐ office work
- ☐ sales assistant
- ☐ restaurant business
- ☐ education
- ☐ health work
- ☐ taxy driver
- ☐ delivery person
- ☐ retail
- ☐ student
- ☐ housemaker
- Do you live with someone over 65 years old?
- ☐ Yes
- ☐ No
- Do you live with children?
- ☐ Yes
- ☐ No
- Do you live with someone who has an underlying illness such as heart disease, respiratory illness, or diabetes?
- ☐ Yes
- ☐ No
- Are you suffering from?
- ☐ Crohn's Disease
- ☐ Ulcerative Colitis
- What is your comorbidity
- ☐ COPD
- ☐ Bronchial asthma
- ☐ Hypertension
- ☐ Diabetes
- ☐ Cardiovascular disease
- ☐ Renal disease
- ☐ Hepatic disorder
- ☐ Psychiatric disorder
- ☐ Others
- ☐ None
- How many years ago were you diagnosed with the disease?

___

- Did you get a COVID-19 infection?
- ☐ No.
- ☐ Yes, mild symptom.
- ☐Yes, moderate symptom.
- ☐Yes, severe symptom.
- Did you have close contact with a person infected with COVID-19?
- ☐ Yes
- ☐ No

• Have you been quarantined for COVID-19?
☐ Yes
☐ No
• Please answer the items you are worried about regarding the COVID-19 vaccine.
☐ Short-term side effects such as allergies
☐ Long-term side effects
☐ Unknown duration of vaccine efficacy
☐ Short period from development to approval,
☐ Vaccine development to approval process is unreliable
☐ Nothing in particular
☐ Others
• Have you been vaccinated against COVID-19?
☐ vaccinated with one dose
☐ vaccinated with two doses
☐ intend to be vaccinated
☐ hesitant to vaccinate
• For those who have been vaccinated or intend to take the COVID-19 vaccine. What is the most applicable reason for choosing vaccination?
☐ Acquiring immunity against COVID-19
☐ Low incidence of adverse effects
☐ Protecting others from COVID-19 infection
☐ Acquiring herd immunity
☐ Increased risk of severe COVID-19 due to old age or comorbidities
☐ Desire to return to normal life
☐ Recommendation from your physician
☐ Receiving immunosuppressive therapy
• For those who do not intend to take vaccine. What is the most applicable reason for choosing not to be vaccinated?
☐ Concerned about short-term adverse reaction.
☐ Concerned that long-term safety of vaccines is unknown.
☐ The period from development to approval is short and unreliable.
☐ Lack of trust regarding vaccine development or testing process.
☐ Vaccine development to approval process is unreliable.
☐ Instruction not to vaccinate by your physician.
☐ Receiving immunosuppressive therapy.
• Do you smoke cigarettes / cigars?
☐ Yes
☐ No
☐ Former smoker
• Do you consume alcohol?
☐ Yes, often or in large quantities
☐ Yes, minimal consumption
☐ NO
• Please tell us the treatment for gastrointestinal disorders that you are currently using?
☐ Mesalazine
☐ Corticosteorides
☐ Azathioprine or 6-Mercaptopurine
☐ anti-TNF products (infliximab (Remicade), adalimumab (Humira), golimumab (Sympony))
☐ Vedolizumab (Entyvio)
☐ Ustekinumab (Stelara)
☐ Tofacitinib (Xeljanz)
☐ Chinese herbal medicine

☐ Enteral nutrition
